# The Insulator Protein SU(HW) Fine-Tunes Nuclear Lamina Interactions of the *Drosophila* Genome

**DOI:** 10.1371/journal.pone.0015013

**Published:** 2010-11-24

**Authors:** Joke G. van Bemmel, Ludo Pagie, Ulrich Braunschweig, Wim Brugman, Wouter Meuleman, Ron M. Kerkhoven, Bas van Steensel

**Affiliations:** 1 Division of Gene Regulation, Netherlands Cancer Institute, Amsterdam, The Netherlands; 2 Division of Molecular Biology, Netherlands Cancer Institute, Amsterdam, The Netherlands; 3 Central Microarray Facility, Netherlands Cancer Institute, Amsterdam, The Netherlands; 4 Delft Bioinformatics Lab, Delft University of Technology, Delft, The Netherlands; Radboud University Nijmegen, The Netherlands

## Abstract

Specific interactions of the genome with the nuclear lamina (NL) are thought to assist chromosome folding inside the nucleus and to contribute to the regulation of gene expression. High-resolution mapping has recently identified hundreds of large, sharply defined lamina-associated domains (LADs) in the human genome, and suggested that the insulator protein CTCF may help to demarcate these domains. Here, we report the detailed structure of LADs in *Drosophila* cells, and investigate the putative roles of five insulator proteins in LAD organization. We found that the Drosophila genome is also organized in discrete LADs, which are about five times smaller than human LADs but contain on average a similar number of genes. Systematic comparison to new and published insulator binding maps shows that only SU(HW) binds preferentially at LAD borders and at specific positions inside LADs, while GAF, CTCF, BEAF-32 and DWG are mostly absent from these regions. By knockdown and overexpression studies we demonstrate that SU(HW) weakens genome – NL interactions through a local antagonistic effect, but we did not obtain evidence that it is essential for border formation. Our results provide insights into the evolution of LAD organization and identify SU(HW) as a fine-tuner of genome – NL interactions.

## Introduction

The nuclear lamina (NL), a dense fibrillar network covering the inside of the nuclear membrane in metazoan cells [reviewed in 1], is thought to represent a major structural element for the nuclear organization of the genome. Close contacts between the NL and chromatin have been observed by electron microscopy [2] and more recently by three-dimensional structured illumination microscopy [3]. Based on FISH studies specific loci are known to preferentially localize at the periphery [reviewed in 4], [5]. Genome-wide mapping using the DamID technology [6] in *Drosophila* Kc cells demonstrated hundreds of genes to be in molecular contact with the NL [7]. These genes are strongly repressed and lack active histone marks. Application of the same mapping technology at a higher resolution in human lung fibroblasts showed that NL interactions occur through large continuous genomic domains with sharply defined borders [8]. In these Lamina Associated Domains (LADs) gene expression is strongly repressed, RNA Polymerase II (RNApolII) and active histone marks are depleted, and repressive histone marks are enriched.

Several observations indicate that LADs are not just passively pushed towards the periphery, but instead are the result of specific NL – genome interactions. Human LAD borders tend to be marked by sequence elements such as outward orientated promoters, CTCF binding sites and CpG islands [8], which indicates that the association with the NL could be controlled by DNA sequence. Furthermore, loss or mutation of lamins in flies and mammals can cause dissociation from the periphery and changes in gene expression, histone modifications and binding of chromatin proteins [9], [10], [11], [12], [13], [14] indicating the functional relevance of genome – lamina associations. In addition, upon differentiation hundreds of genes move from or towards the NL, correlating with their respectively increased and decreased expression levels [7], [15], [16]. Taken together these point to the existence of mechanism that regulate genome-NL interactions.

The current knowledge about such a regulatory mechanism is limited. Repressive histone marks are most likely not involved, since loss of histone methylatransferases or DNA methylatransferases do not effect peripheral localization of single loci [15], [17]. Possibly the absence of active histone marks could play a role, since treatment with an HDAC inhibitor has been shown to disrupt molecular interactions with the NL in *Drosophila* Kc cells [7] and to cause dissociation of genes from the nuclear periphery in mammalian cells [18]. Alternatively, DNA-binding proteins that physically interact with the NL could be involved in modulating NL interactions.

Because a subset of human LAD borders is marked by an insulator element, the CTCF binding sequence [8], we reasoned that insulator proteins are likely candidates to be involved in modulating genome-NL interactions. Insulator elements are DNA sequences that, when bound by insulator proteins, are thought to play a key role in chromatin organization by mediating intra- and interchromosomal interactions. They have been shown to block the communication between a promoter and its enhancer when placed in between [19], [20], [reviewed in 21] and some insulators are thought to separate inactive from active chromatin domains by acting as a barrier against spreading of repressive chromatin proteins or histone modifications into neighbouring regions [reviewed in 21], [22].

In *Drosophila* five main insulator proteins have been identified; Suppressor of Hairy-wing (SU(HW)), CCCTC-binding Factor (CTCF), Boundary Element-associated Factor (BEAF-32), Zeste-white 5 (Zw5) -also known as Deformed Wings (DWG)- and GAGA Factor (GAF) [19], [23], [24], [25], [26], [27], [reviewed in 28]. Especially SU(HW) is a promising candidate to regulate genome – NL interactions since the SU(HW) complex member TOPORS is shown to interact with lamin proteins [29].

Here we analyze the possible roles of insulator proteins in the regulation of NL- genome interactions in *Drosophila* Kc cells. We report a high resolution map of *Drosophila* genome – NL interactions, showing that *Drosophila* LADs exhibit remarkably similar characteristics as their human counterparts. Comparison to new and published binding maps for all five insulators revealed SU(HW) to be the only insulator protein that preferentially binds at LAD borders and at specific positions inside LADs. By direct functional studies we demonstrate that SU(HW) modulates LAD – NL interactions through a local antagonistic effect. We thus identified SU(HW) as the first protein to fine-tune molecular interactions between the genome and the NL.

## Results

### LADs in the *Drosophila* genome

A previous DamID study in *Drosophila* Kc cells identified hundreds of genes that associate with the NL [7]. However, this study lacked the resolution required for a detailed view of genome - NL interaction patterns. We therefore repeated these DamID experiments for LAM (also known als Lamin-Dm0, the only B-type lamin in *Drosophila*), this time using a high-density microarray that queried the entire fly genome with a median probe spacing of ∼300 bp. With DamID, DNA adenine methyltransferase (Dam) fused to LAM leaves a stable adenine-methylation ‘footprint’ *in vivo* at the interaction sites. Previous comparisons to fluoresence in situ hybridization data have indicated that DamID signals obtained with LAM can be interpreted as relative molecular contact frequencies between the NL and the probed genomic locus [7], [8]. Note that the Dam-LAM fusion protein is expressed at very low levels, preventing overexpression artifacts.

**Figure 1:**
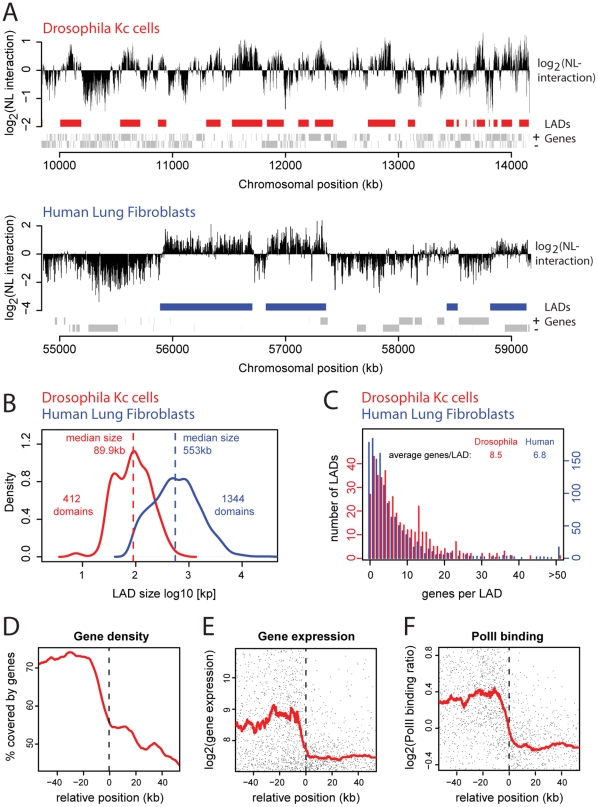
Lamina associated domains in the *Drosophila* genome. (**A**) Genome – NL interaction maps in *Drosophila* Kc cells and human Lung Fibroblasts along a 4 Mb region at respectively chromosome 2L and chromosome 18. Human data are from [8]. Y-axes depict the log_2_ transformed Dam-LAM over Dam-only methylation ratio, smoothed by a running median of respectively 15 and 5 probes. Rectangles below each map represent calculated LAD positions for *Drosophila* (red) and human (blue). Grey rectangles at the bottom represent genes at the + and - strand. (**B**) Distribution of LAD sizes in *Drosophila* (red) and human cells (blue). Dashed lines mark the median LAD sizes. (**C**) Histogram of the number of genes per LAD. (**D–F**) Profiles across aligned LAD borders (824 borders, left and mirrored right borders combined). Running window median (red line) and a random subset of 2001 single genes (black dots in E and F). The region around each border from which data was taken ranges from the center of the inter-LAD region to the center of the LAD; this ensures that each data point is used only once. X-axis depicts the position relative to the nearest LAD border; positive coordinates inside, negative coordinates outside LADs. (**D**) Median gene coverage. (**E**) mRNA levels in A-values, (log_2_(Cy5)+log_2_(Cy3))/2 (**F**) Median RpII18 occupancy on entire genes as determined by DamID [data from30].

We averaged the data of two independent DamID experiments, which highly correlated with each other (Pearson correlation of 0.77) and with the previously published low resolution data (Pearson correlation of 0.74). The resulting profile (Fig. 1a, Fig. S1) shows that the genome in Kc cells is associated with the NL through large continuous domains, alternating with regions of low association. A domain detection algorithm, previously developed for the analysis of human NL interaction data [8] identified a total of 412 *Drosophila* Lamina Associated Domains (LADs) (Table S1). These LADs vary in size between 7 and 700 kb, with a median size of ∼90 kb (red line in Fig. 1b). In total they cover 40% of the genome (data not shown).

The *Drosophila* genome is much more compact than the human genome. Although the overall gene counts differ by only 2-fold (respectively 14,449 and ∼31,000 genes), the *Drosophila* genome is ∼25 times smaller, and *Drosophila* genes are typically shorter and more closely spaced than human genes. These differences in scale raise the question whether the organization of the *Drosophila* genome in LADs also occurs at a smaller scale. Indeed, human LADs are much bigger, ranging from ∼0.1 Mb to ∼10 Mb with a median LAD size of 553 kb (blue line in Fig. 1b) [8]. However, we find that the number of genes per LAD is remarkably similar between the two species, on average respectively 6.8 and 8.5 genes/LAD in human and *Drosophila* cells (Fig. 1c). These observations suggest that LAD organization has co-evolved with the linear spacing and size of genes, and that the number of genes, rather than the absolute length of DNA, is an important structural parameter of LADs. Taken together, these results demonstrate that the organization of the genome into LADs is conserved between *Drosophila* and human cells, albeit at different scales.

### *Drosophila* LADs are repressed chromatin domains

Human LADs represent a repressive chromatin environment with a relatively low gene density. To investigate whether *Drosophila* LADs exhibit similar characteristics, we aligned the 412 LADs by their left as well as their mirrored right borders and calculated the average profiles for several features across the 824 combined borders. This analysis revealed that 45–55% of the sequence within LADs consists of genes, while outside LADs the gene coverage is ∼70% (Fig. 1d).

In total, the 412 LADs in Kc cells contain about 30% of all genes. To assess the expression status of these genes we measured the mRNA expression levels of nearly all genes in Kc cells using microarrays and calculated the expression profile across the aligned LADs. As is the case for human LADs, almost all genes inside LADs are expressed at baseline levels, while genes outside LADs display varying and on average higher expression levels (Fig. 1e). Consistent with this, the binding of the 18-kDa subunit of RNA polymerase (RpII18) to genes [30] shows a low median level and a low variance inside LADs, compared to inter-LAD regions (Fig. 1f). The median mRNA expression levels and RpII18 binding levels both exhibit a sharp transition at LAD borders (red lines in Fig. 1e and 1f), similar to what has been reported for human LADs [8]. Taken together, *Drosophila* LADs exhibit similar characteristics as their human counterparts: they represent a repressive type of chromatin with sharp transitions.

### Genome-wide identification of *in vivo* binding sites of insulator proteins

**Figure 2:**
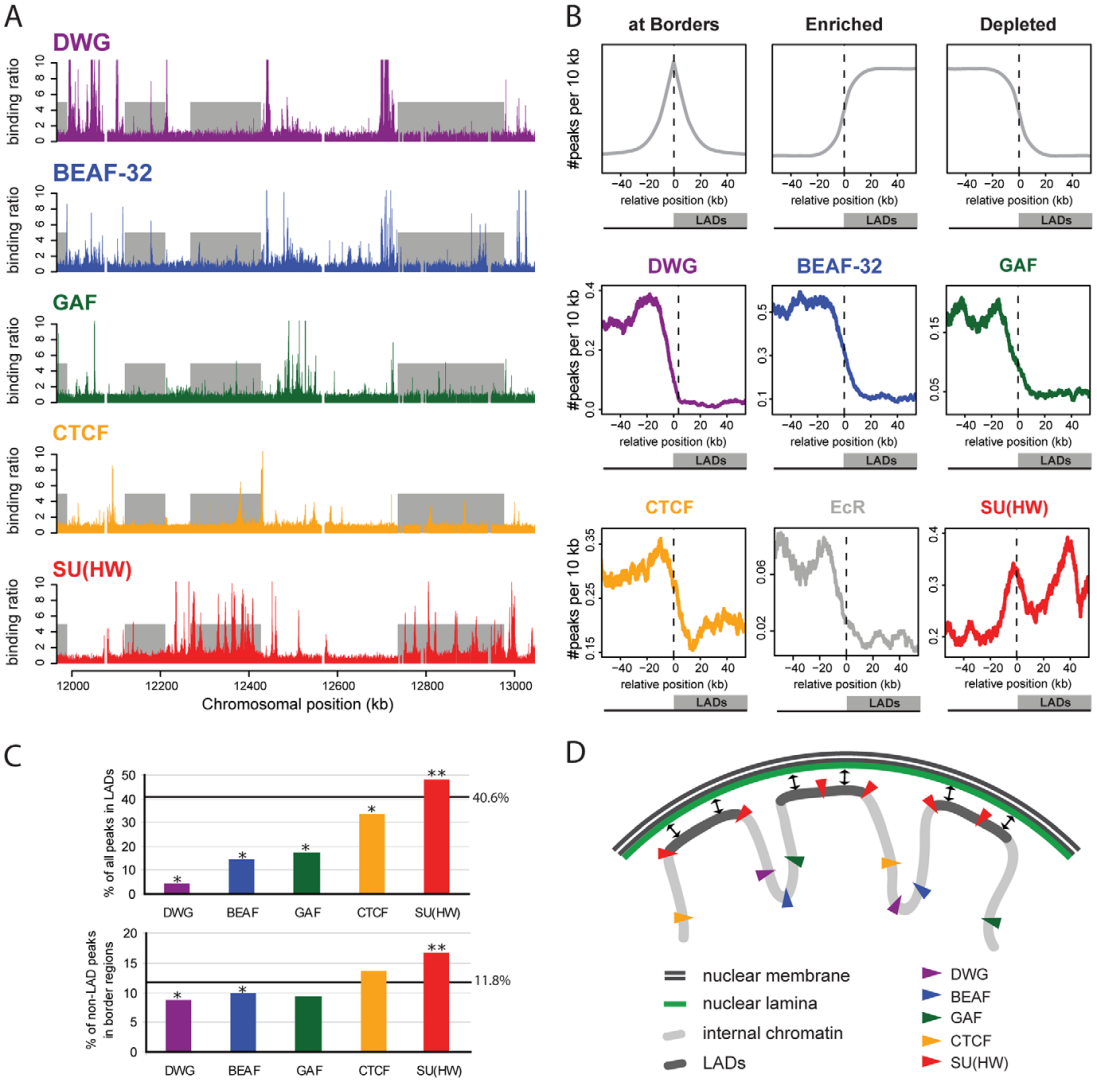
SU(HW) sites are enriched at LAD borders and within LADs. (**A**) Binding maps of insulator proteins along a 1 Mb region on chromosome 2L. Y-axes depict the Dam-insulator over Dam-only methylation ratio (high values are truncated at 10). Grey rectangles represent LADs. (**B**) Theoretical profiles of features that are respectively enriched at LAD borders, enriched inside LADs or depleted from LADs (upper panels). Profiles of insulator and EcR binding peaks across aligned LAD borders. 824 borders, left and mirrored right borders combined (lower panels). The region around each border from which data was taken ranges from the center of the inter-LAD region to the center of the LAD; this ensures that each data point is used only once. X-axis depicts the position relative to the nearest LAD border; positive coordinates inside LADs and negative coordinates outside LADs. Y-axes depict the median number of binding peaks within a running window of 10 kb. Y-axes are scaled to frequencies within the plotted window, depending on the genome-wide frequency of the feature. (**C**) Percentage of insulator binding peaks within LADs (*top*) and percentage of inter-LAD peaks within a 10 kb region just outside LADs (*bottom*). Black horizontal lines represent the percentage expected by chance. **significantly enriched or *depleted compared to random permutation simulations; p<10^−3^. (**D**) Model of the chromatin organization in LADs, with SU(HW) binding mainly at LAD borders and inside LADs; and DWG, BEAF-32, GAF and CTCF preferentially located in inter-LAD regions.

Next, we investigated whether specific insulator proteins are involved in regulating LAD formation. Such an activity requires the candidate insulator protein to bind either inside LADs or at LAD borders. We therefore conducted DamID experiments in Kc cells to obtain whole-genome binding maps for the five known *Drosophila* insulator proteins: BEAF-32, CTCF, DWG, GAF and SU(HW). Full-genome binding profiles of DWG and GAF were previously not available for *Drosophila* Kc cells. Although for CTCF, SU(HW) and BEAF-32 such maps have been reported [31], a proper comparison requires that all the insulator profiles are obtained from the same cells and under the same experimental conditions. For each insulator protein we performed two independent DamID experiments, which highly correlated with each other (Pearson correlation coefficients between 0.71 and 0.83) and with previously published low resolution DamID data (Pearson correlation coefficients of 0.64 for GAF [32] and 0.70 for SU(HW) and BEAF32 [33]). We averaged the duplicate datasets to obtain a single full-genome profile for each protein. The resulting binding profiles of all five insulator proteins are generally characterized by sharp peaks of local enrichment (Fig. 2a, Fig. S2a). A peak detection algorithm (see Methods) identified 2,173 peaks for DWG; 4,027 for BEAF-32; 1,290 for GAF; 2,930 for CTCF; and 2,986 for SU(HW) within the *Drosophila* genome. Each insulator protein has a unique binding pattern, and co-occurrence of different insulators is present but relatively rare (Fig. S2b). An exception is formed by DWG and BEAF-32, which exhibit substantial overlap in their binding pattern.

To validate the insulator DamID profiles we first compared four of the profiles with recent chromatin immunoprecipitation (ChIP) data [31], [34]. This shows that most of the insulator binding peaks detected with DamID coincide with corresponding ChIP peaks (Fig. S3a). As a second and independent validation method we compared the DamID profiles of SU(HW), CTCF and GAF with the occurrence of their cognate DNA binding motifs. The high co-occurrence of DamID binding sites with the corresponding DNA binding motifs (Fig. S3b) further validates the generated profiles. For DWG, ChIP data and a DNA binding motif have not been published before, but the overlap of the DWG and BEAF-32 binding profiles (Fig. 2a, Fig. S2b) is consistent with the reported direct interaction between DWG and BEAF-32 *in vivo* [35]. We conclude that we have successfully generated high-resolution genome-wide binding profiles for five insulator proteins, together with the NL interaction profile, in one and the same *Drosophila* cell type.

### SU(HW) sites are enriched at LAD borders and within LADs

Next, we compared insulator binding profiles with the LAD pattern. Visual inspection led to two observations. First, SU(HW) frequently binds at multiple sites within LADs, while the other four insulator proteins mainly bind outside LADs (Fig. 2a, Fig. S2a). Second, many LAD borders coincide with a binding peak of one of the insulator proteins.

To address whether these observations generally apply at a genome-wide level, we calculated the density of insulator binding peaks across the 824 aligned LAD borders (Fig. 2b). Hypothetically an enrichment at LAD borders or inside LADs would result in respective profiles as depicted in the first two upper panels. The profile of a protein that is depleted from LADs (which would not be expected to be directly involved in LAD formation) is expected to yield a profile as depicted in the third panel. DWG, BEAF-32, CTCF and GAF fall into this latter class: they show similar overall profiles, with the majority of binding sites occurring outside LADs. Their binding frequency gradually decreases across the LAD borders and does not show a peak at the borders themselves, indicating that these four proteins are not specifically enriched at LAD borders. For comparison, the Ecdyson Receptor (EcR) [36], a transcription factor not expected to be linked to NL interactions, yields a similar depletion from LADs. Thus, DWG, BEAF-32, CTCF and GAF are unlikely to play prominent roles in the direct regulation of LAD – NL interactions.

In contrast, SU(HW) exhibits a distinct profile with two prominent regions of enrichment. First, SU(HW) binding peaks are preferentially located in the vicinity of LAD borders, with the highest frequency occurring just outside LADs, at ∼4 kb from the borders. Second, the profile confirmed the visually observed enrichment of SU(HW) binding peaks inside LADs. Strikingly, within LADs the SU(HW) binding peaks are not equally distributed, but instead are concentrated at a distance of ∼40 kb from the nearest LAD border (see below). This occurrence of a SU(HW) peak within the 35–45 kb region from one or the other border is found in a substantial part (51%) of the LADs of which half the size is at least 45 kb (data not shown). Performing the same alignment analysis with ChIP-defined insulator binding peaks [31] results in similar patterns, thereby confirming the validity of our findings with data from an independent experiment and technique (Fig. S3c).

Further statistical analyses confirmed the enrichment of SU(HW) and the depletion of the other four proteins in LADs. For DWG, BEAF-32, CTCF and GAF respectively 4.6%, 14.3%, 17.5% and 33.7% of the binding peaks are located within LADs, while 40.6% is expected by chance. In contrast, 48.1% of the SU(HW) peaks are located within LADs, which is more than expected by chance (top panel Fig. 2c). Statistical testing against random permutation simulations showed that this enrichment of SU(HW) and the depletion of the other insulators in LADs is significant (p<10^−3^). Furthermore, ∼17% of the SU(HW) binding peaks outside LADs are located within border regions (defined as the 10 kb areas just outside LADs), which is a statistically significant enrichment (Statistical testing against random permutation simulations: p<10^−3^) (bottom panel Fig. 2c). None of the four other insulator proteins are significantly enriched in LAD border regions (Statistical testing against random permutation simulations p>0.01). DWG and BEAF-32 are even significantly depleted from border regions (p<10^−3^) while GAF and CTCF bind randomly in LAD border regions, as would be expected by chance. In total, 77% of the LADs and 27% of the LAD border regions contain a least one SU(HW) binding peak.

To address whether SU(HW) is specifically enriched inside LADs and not just in repressive chromatin in general, we compared the SU(HW) binding peaks to Polycomb-bound regions (Fig. S4), which are large chromatin domains that are mostly transcriptionally inactive [37] and only partly overlap with LADs. The amount of overlap between Polycomb domains and LADs is roughly as may be expected by random chance, namely 40%. Statistical analysis revealed that, in contrast to LADs, Polycomb domains are significantly depleted of SU(HW) binding sites. We find 4.9% of the SU(HW) peaks to be located within Polycomb domains, while 9.3% is expected by chance, showing that the enrichment of SU(HW) is specific for NL-interacting chromatin.

Taken together, these results demonstrate that SU(HW) binding is significantly and specifically enriched just outside LAD borders, as well as at specific locations within LADs (red triangles in Fig. 2d). Furthermore, even though DWG, BEAF-32, dCTCF and GAF binding sites occasionally overlap with individual LAD borders, they do not show a statistically significant global preference for LADs or LAD borders (colored triangles in Fig. 2d).

### Enrichment of SU(HW) is sequence driven

**Figure 3:**
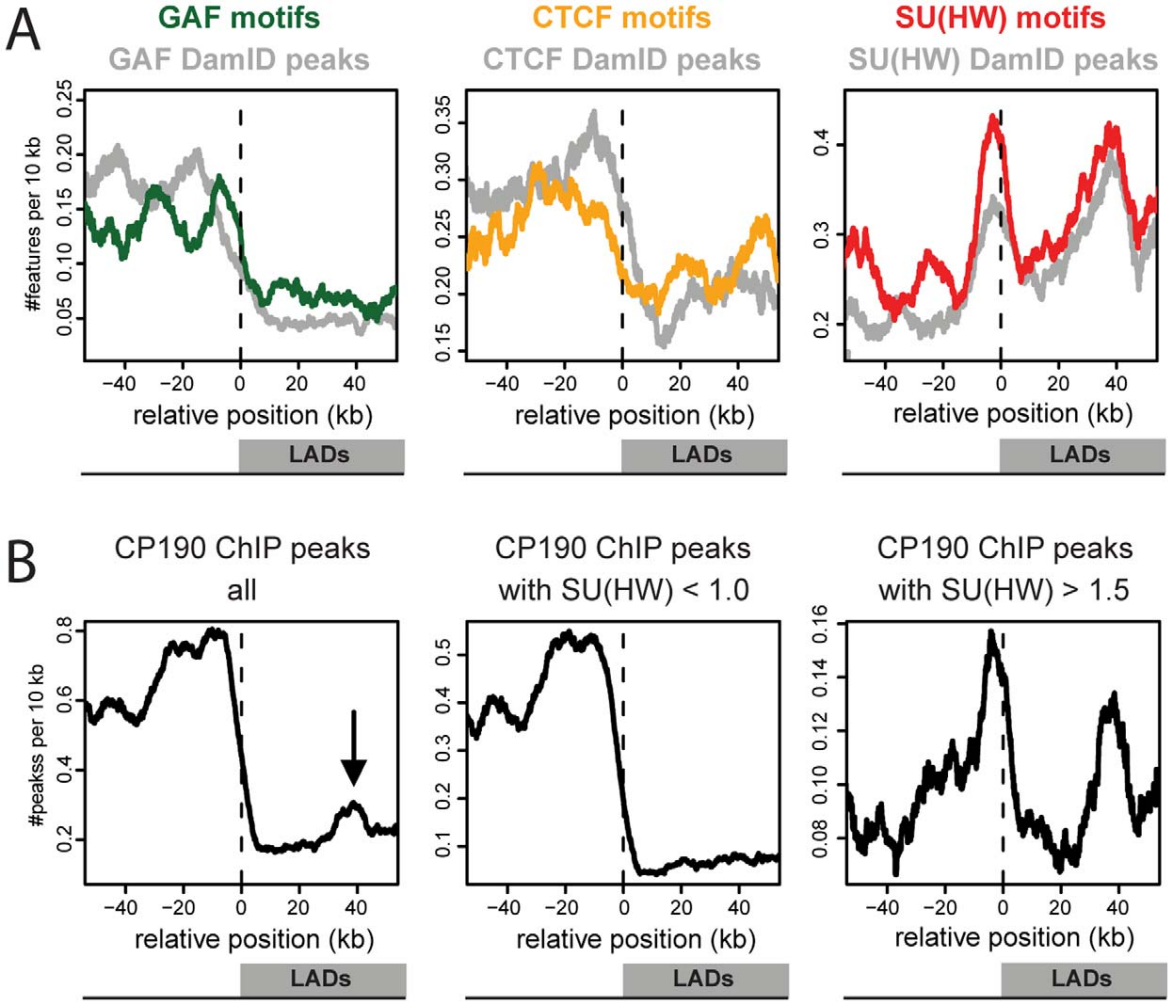
SU(HW) distribution relative to LADs is sequence driven and linked to CP190 binding. (**A**) Profiles of sequence motifs across aligned LAD borders. X-axis depicts the position relative to the nearest LAD border; positive coordinates inside LADs and negative coordinates outside LADs. Colored lines show the median frequency within a running window of 10 kb for sequence motifs, grey lines for DamID identified peaks. (**B**) Profiles of CP190 peaks [28] across aligned LAD borders; all Cp190 peaks (1^st^ panel), CP190 peaks without SU(HW) binding, defined as peaks with an average log_2_(SU(HW)binding ratio) <1.0 (2^nd^ panel), CP190 peaks with SU(HW) binding, defined as CP190 peaks with an average log_2_(SU(HW)binding ratio) >1.5 (3^rd^ panel).

The observed enrichment of SU(HW) and the paucity of the other four insulator proteins in LADs could be dictated by the genomic distribution of the corresponding DNA binding motifs, which is likely since the presence of a SU(HW) DNA binding motif is known to be highly predictive for SU(HW) binding [38]. Alternatively, the patterns could be driven by respectively cooperative or exclusive interactions with other chromatin components in LADs. To discriminate between these two mechanisms we compared the occurrence of protein binding peaks to the distribution of the corresponding sequence motifs for CTCF, GAF and SU(HW) (respectively grey and colored lines in Fig. 3a). This revealed that the distributions of these three insulator proteins and their cognate motifs are highly similar when aligned to LADs and LAD borders. The motifs of CTCF and GAF are depleted in LADs and not specifically enriched in border regions. In contrast, the motif of SU(HW) is enriched within LADs as well as in border regions. Importantly, the SU(HW) motif shows the same prominent enrichment inside LADs at ∼40 kb from LAD borders as was observed for SU(HW) binding. Thus, the enrichments of SU(HW) at LAD borders and at the +40 kb position are to a large extent “hard-coded” in the sequence of the *Drosophila* genome.

The enrichment of SU(HW) at +40 kb can not be explained by a subgroup of specifically sized LADs, since alignments of subgroups of differently sized LADs all result in an enrichment at ∼40 kb (data not shown). The enrichment of SU(HW) at +40 kb is also not caused by a genome-wide periodicity of SU(HW) binding itself: a histogram of the pair-wise distances between all SU(HW) peaks shows no preferential spacing of SU(HW) peaks in the range of 40 kb (Fig. S5). In addition, we found no significant correlation between the presence of SU(HW) at a LAD border and binding of SU(HW) in the same LAD around +40 kb (Fisher's exact test p>0.1, data not shown). Thus, SU(HW) shows preferences for LAD borders as well as for the +40 kb position within LADs, but these SU(HW) binding events appear not to be linked. In summary, these results show that the remarkable pattern of SU(HW) relative to LADs is driven by the distribution of its binding motif in the genome.

Enrichment of SU(HW) containing CP190 peaks indicates functionality.

To further investigate the remarkable pattern of SU(HW) relative to LADs we analysed the distribution across aligned LAD borders of CP190 binding peaks (defined with ChIP by Bushey et al. [31]). CP190, a subunit of different insulator protein complexes, is thought to be necessary for insulator function since it is essential for both insulator body formation and enhancer blocking activity [31], [39], [40], [41]. Figure 3b (1^st^ panel) shows that CP190 is mostly bound outside LADs. Interestingly, the profile also exhibits a modest local peak of enrichment inside LADs, exactly at ∼40 kb from the LAD borders. CP190 insulator complexes can be divided in at least three different subclasses, containing either SU(HW), CTCF or BEAF-32 [31]. The profile of CP190 binding peaks across LAD borders can therefore be subdivided in peaks that co-localize with SU(HW) and peaks that do not (Figure 3b, 2^nd^ and 3^rd^ panels). Remarkably, CP190 binding peaks that do not contain SU(HW) binding (defined as CP190 peaks with an average log_2_(SU(HW)binding ratio) <1) are depleted from LADs, without any enrichment at +40 kb. They probably represent the BEAF-32 and CTCF containing subclasses of CP190 binding peaks. Strikingly, the Su(Hw) containing subclass (defined as CP190 peaks with an average log_2_(SU(HW)binding ratio) >1.5) strongly resembles the profile of SU(HW) itself, showing the enrichment just outside LAD borders as well as at +40 kb. Taken together, the enrichment of SU(HW) at LAD borders as well as at 40 kb inside LADs is confirmed by the binding of CP190, and thus is likely to involve functional insulator protein complexes.

### SU(HW) binding antagonizes genome - NL interactions

The surprising pattern of SU(HW) binding relative to LADs suggested two possible roles for SU(HW) in the regulation of genome - NL associations. First, the binding of SU(HW) at LAD borders could help to separate LADs from inter-LADs. Second, the SU(HW) binding inside LADs could modulate NL interactions of LADs. To directly test these hypotheses, we monitored the genome-wide changes in NL interactions after alteration of the expression level of SU(HW). Specifically, we either reduced SU(HW) levels by RNA interference (RNAi), or we increased SU(HW) levels by transfection with a SU(HW) expression vector. We then created new full-genome DamID maps of NL interactions.

**Figure 4:**
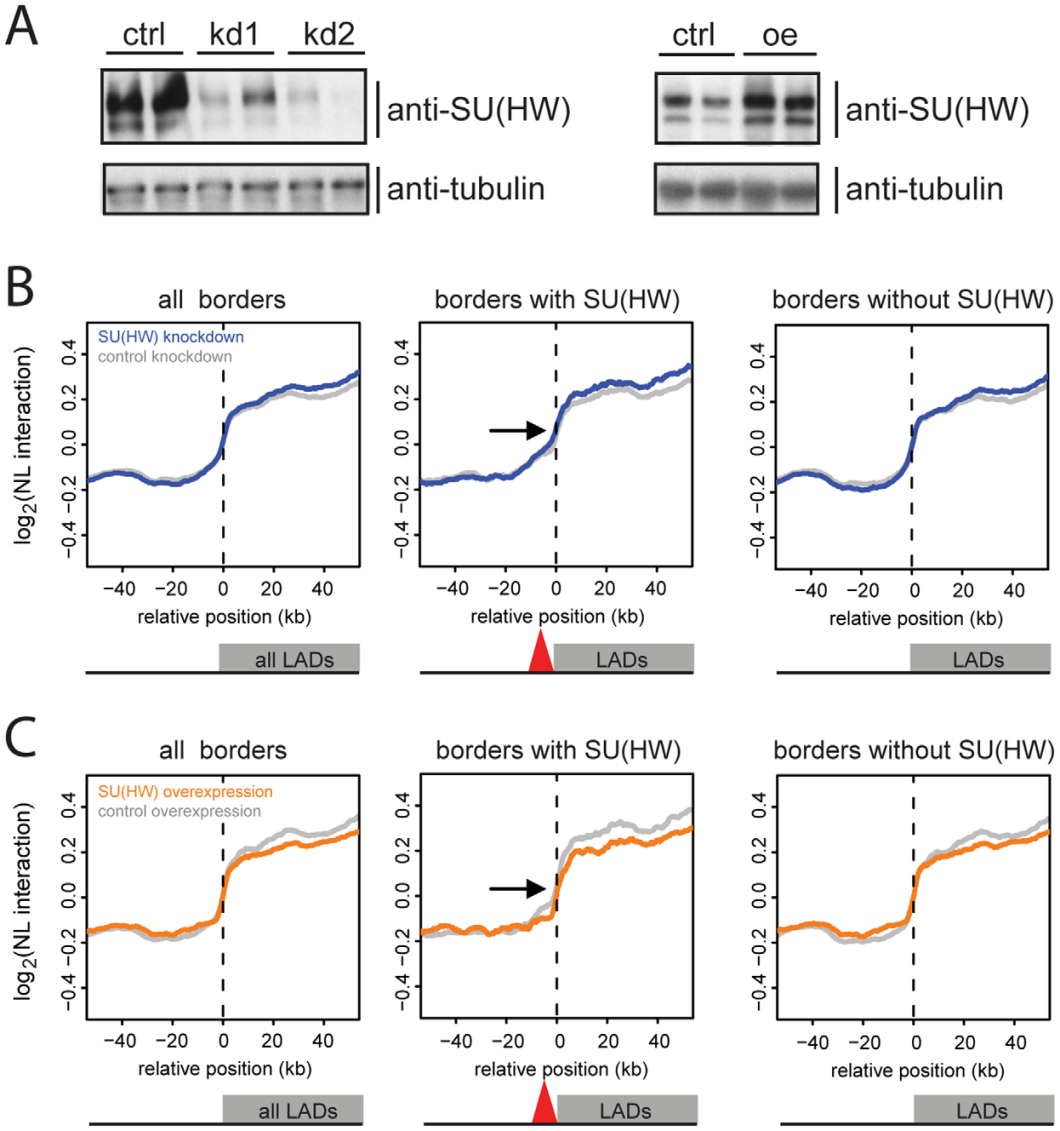
SU(HW) alone is not essential for demarcation of LAD borders. Genome - NL interaction maps after knockdown and overexpression of SU(HW). (**A**) Western blot analysis of SU(HW) expression levels after knockdown (ctrl: control RNAi; kd1 and kd2: SU(HW) RNAi with two independent dsRNA fragments) and after overexpression (ctrl: control vector oe: overexression by transfection of SU(HW) under an Act5C promoter). 1^st^ lane in each panel: transfected with Dam-LAM, 2^nd^ lane with Dam-only. (**B–C**) Median NL interaction (log_2_ Dam-LAM/Dam ratio) across all aligned LAD borders (824 borders, 1^st^ panel); border regions with SU(HW) present (220 borders, 2^nd^ panel, red triangle represents SU(HW) at the borders), borders without SUH(HW) present (604 borders, 3^rd^ panel). (**B**) Knockdown of SU(HW) (blue line) and control knockdown (grey line). (**C**) Overexpression of SU(HW) (orange line), and corresponding control (grey line).

For RNAi we used two different, non-overlapping, double-stranded RNA (dsRNA) fragments to exclude off-target effects. Treatment with a dsRNA fragment derived from the unrelated *white* gene served as a control. Western blot analysis showed that both *su(Hw)* dsRNA fragments caused efficient knockdown of the SU(HW) protein (Fig. 4a, 1^st^ panel). Knockdown of SU(HW) had no effect on the doubling time of the cells (data not shown), ruling out secondary effects of an altered cell cycle on the DamID pattern. Elevated levels of SU(HW) were obtained by co-transfection of the DamID plasmids with a vector that drives expression of SU(HW) from an *Act5C* promoter. Western blot analysis showed only a slight increase in expression of SU(HW), presumably because only a minority of cells is transfected (Fig. 4a, 2^nd^ panel). However, because the overexpression vector is co-transfected with the DamID vector, overexpression may be expected to be more prominent in cells that express Dam-LAM. We generated DamID maps of NL association for each treatment in two independent experiments.

If SU(HW) is involved in the demarcation of LAD borders, we reasoned that loss of SU(HW) would lead to changes in NL interactions near the borders where SU(HW) is bound, such as expansion or contraction of LADs, or decreased sharpness of borders. However, the median NL interaction profile across LAD borders with SU(HW) bound within 10 kb outside the LAD showed no dramatic change at the borders: the median curve did not shift laterally, nor did it change in steepness when comparing SU(HW) knockdown to control cells (blue vs grey in Fig. 4b). Overexpression of SU(HW) also had no detectable effect on LAD border sharpness or position (orange vs grey in Fig. 4c). We therefore conclude that under the conditions and in the cell type tested, SU(HW) is not essential for the demarcation of LAD borders, despite the clear enrichment of SU(HW) binding sites at LAD borders.

**Figure 5:**
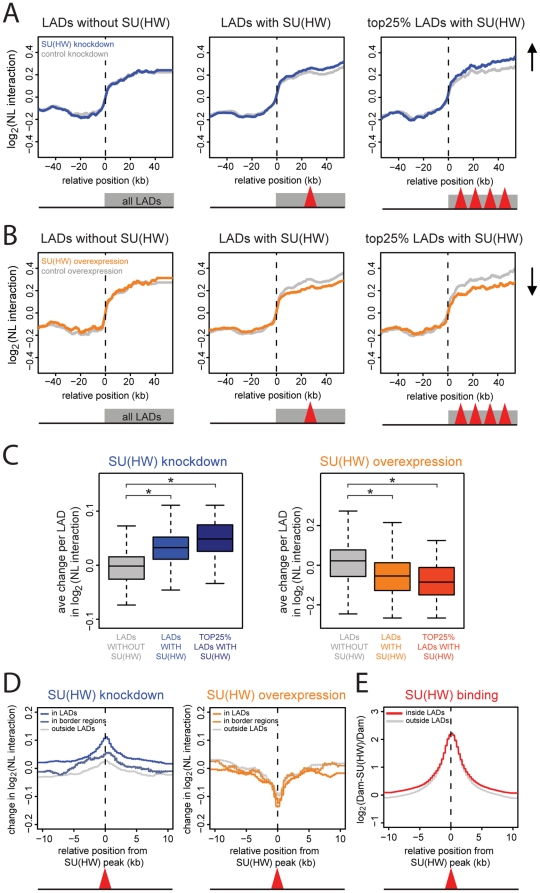
SU(HW) is a local antagonist of genome – NL interactions. (**A–B**) Median NL interaction (log_2_ Dam-LAM/Dam ratio) across LADs without SU(HW) peaks (190 borders, 1^st^ panel), LADs with at least one SU(HW) peak (634 borders, 2^nd^ panel, red triangle represents the presence of one or more SU(HW) peaks at any position inside LADs), the 25% of LADs with the highest SU(HW) peak density (206 borders, 3^rd^ panel, red triangles represent high density of SU(HW) peaks). (**A**) After knockdown of SU(HW) (blue line), after control knockdown (grey line). (**B**) After overexpression of SU(HW) (orange line), after control overexpression (grey line). (**C**) Ave changes in NL interaction levels per LAD, for LADs without SU(HW) (grey), LADs with at least one SU(HW) peak (light blue or orange), the 25% of LADs with the highest SU(HW) peak density (dark blue or orange) after knockdown (blue, 1^st^ panel) and overexpression of SU(HW) (orange, 2^nd^ panel). * Wilcoxon test; p<10^−3^ (**D**) Median changes in NL interaction across aligned SU(HW) peaks (red triangle) inside LADs (bright lines), in border regions (pale lines) and outside LADs (grey lines) after knockdown of SU(HW) (blue, 1^st^ panel) and after overexpression of SU(HW) (orange, 2^nd^ panel). (**E**) *Cis*-spreading of SU(HW) DamID signals from aligned SU(HW) binding peaks (red triangle), inside LADs (red line) and outside LADs (grey line).

In contrast, we did notice effects of altered SU(HW) levels on genome - NL interactions inside LADs. Knockdown of SU(HW) caused a specific increase in NL interaction levels inside LADs, while overexpression of SU(HW) had the opposite effect inside LADs (color vs grey in the 1^st^ panels of Fig. 4b,c). Thus, SU(HW) antagonizes genome – NL interactions inside LADs. To confirm the specificity of this effect, we repeated this analysis after dividing LADs into three classes: LADs without SU(HW) binding peaks inside, LADs with at least one SU(HW) binding peak inside and the 25% of LADs with the highest SU(HW) peak density (Fig. 5a,b). This shows that the effects of altered SU(HW) expression levels are restricted to LADs that harbor SU(HW) binding sites, and that the overall effect is proportional to the density of SU(HW) peaks.

To visualize this dependency further we calculated the average change in genome – NL interactions per LAD (Fig. 5c), showing that the change in NL interactions is indeed increasing with higher SU(HW) peak density. A Wilcoxon test between the grouped LADs confirmed NL interactions to significantly increase and decrease after respectively knockdown and overexpression of SU(HW) (p<10^−3^). Repeating this analysis for the individual replicates showed the same trend (Fig. S6), thereby confirming the reproducibility of the observed changes in genome – NL interactions.

Together, these results demonstrate that SU(HW) reduces the frequency of NL interactions inside LADs.

The observation that only LADs with SU(HW) binding sites are affected by changes in SU(HW) levels indicates that SU(HW) controls genome – NL interactions locally rather than globally. To investigate the range over which SU(HW) acts *in cis*, we plotted the change in NL interactions as a function of the distance to the nearest SU(HW) binding site, after knockdown and overexpression of SU(HW) (Fig. 5d). This reveals that the antagonistic effect of SU(HW) on NL interactions is most pronounced within ∼5 kb from the SU(HW) binding sites, but extends to >10 kb. A weaker effect is also seen at SU(HW) sites in border regions and outside LADs, indicating that in these regions SU(HW) may help to suppress spurious contacts with the NL. Interestingly, the range of the SU(HW) effects on NL interactions corresponds approximately to the width of the SU(HW) binding peaks (Fig. 5e). Moreover, an elevated baseline of SU(HW) interactions, extending over >10 kb from the binding peak centers, is present specifically inside LADs. Thus, the *cis*-effect of SU(HW) on NL interactions has a similar distribution as the actual contacts of SU(HW) with sequences surrounding the SU(HW) binding sites. Taken together, our data reveal that SU(HW) binding antagonizes genome - NL interactions in a local fashion, over a distance that roughly corresponds to the range over which SU(HW) contacts the genome.

## Discussion

Here, we report the fine structure of LADs in a *Drosophila* cell line, and analyze the genome-wide distribution of five insulator proteins with respect to LADs. The DamID method is particularly suited for the detailed mapping of NL interactions. DamID offers molecular resolution that cannot be obtained by traditional microscopy-based methods such as fluorescent in situ hybridization. This allowed us to compare NL interactions and insulator protein binding sites at a resolution of ∼1 kb. We discovered that SU(HW) is the only tested insulator protein that preferentially interacts with DNA in LADs and at LAD borders. Furthermore, we demonstrate that SU(HW) modulates genome – nuclear lamina (NL) interactions by local antagonism inside LADs. Because interactions as detected by DamID require at least transient physical contact of the Dam-Lam protein with the chromatin fiber, we interpret changes in DamID signals as local changes in the molecular contact frequency between the NL and the probed genomic locus.

### SU(HW) as an antagonist of LAD – NL interactions

The found enrichment of SU(HW) in LADs supports previous ideas about the peripheral localization of SU(HW). SU(HW) is thought to form large aggregates that are often located at the nuclear periphery [42] and a SU(HW) protein complex member, TOPORS, is shown to interact with the NL. The association with the NL seems to be essential since the enhancer blocking activity of the *gypsy* insulator is disturbed by a *lamin* mutation [29]. Our data demonstrate that SU(HW) has an inhibitory effect on genome – NL interactions. This seems at odds with previous observations that the *gypsy* transposable element, which harbors a short array of SU(HW) binding motifs, can target flanking DNA to the nuclear periphery in a SU(HW) – dependent manner [43]. Possibly, the effect of SU(HW) on the nuclear location of *gypsy* is different from that on most LADs, perhaps due to a different sequence context. It is also possible that unknown factors cause SU(HW) to switch from an antagonist to an agonist of LAD – NL interactions depending on the cell type.

At present, it is not known how LADs associate with the NL. Still, we can envision several mechanisms for the antagonistic action of SU(HW). For example, SU(HW) could form bulky DNA-associated complexes that locally disrupt NL interactions, which would be supported by the believe that SU(HW) forms aggregates at the nuclear periphery [42]. In addition distant *gypsy* elements have been suggested to interact with each other [44], [45] to form chromatin loops in a SU(HW) dependent manner [46]. Hence, if SU(HW) promotes associations between a LAD and a locus located in the nuclear interior, then naturally the LAD would be less frequently located at the NL.

The presence of a protein inside LADs that antagonizes LAD – NL interactions is somewhat paradoxical. We propose that SU(HW) is important for the fine-tuning of NL interactions. For example, by loosening of LAD – NL associations, SU(HW) may facilitate switches in NL associations of some loci during cellular differentiation. In this respect it is interesting to note that SU(HW) is expressed at particularly high levels in embryos and pupae (http://flybase.org/reports/FBgn0003567.html), when many cells differentiate into new cell types, while the expression is low in larvae and adult flies when relatively few cells change their identity. In addition a fraction of the SU(HW) binding sites has been found to be cell-type specific [31]. Although the antagonistic effect of SU(HW) appears relatively modest at individual loci, the large numbers of SU(HW) sites in many LADs may together modulate chromosome organization to a significant degree.

### Evolutionary aspects of LADs and their borders

Our high-resolution map of NL interactions reveals that the fly genome is organized into hundreds of discrete LADs, similar to the human genome. Like in human LADs, most genes in fly LADs exhibit low occupancy by the RNA polymerase II 18 kD subunit (RPII18) and they are expressed at low levels. Even though *Drosophila* LADs are about five-fold smaller than human LADs, the number of genes per LAD is remarkably similar between the two species. This suggests that LAD organization has co-evolved with the linear spacing and size of genes along the two genomes.

Human and fly LADs are also similar in their demarcation of borders by specific insulator proteins. Surprisingly, the two species employ different insulators for this purpose. In human fibroblasts, a substantial amount of LAD borders is marked by CTCF. In *Drosophila* cells, we observed no such enrichment of CTCF; instead SU(HW) is enriched at LAD borders. SU(HW) is an insect–specific protein [47]. The switch of insulator protein utilization at LAD borders between the two species is remarkable, because this involves not only a functional switch of the two proteins, but simultaneously the co-evolution of the respective binding motifs at many LAD borders.

While the enrichment of SU(HW) near LAD borders is clearly non-random and sequence-based, we were unable to detect significant consequences of the loss or overexpression of SU(HW) on genome – NL interactions around LAD borders. Since SU(HW) alone is not essential for demarcation of LAD borders, we suggest that SU(HW) is redundant with one or more of its partner proteins, such as CP190 or Mod(mdg4) [39], [48]. It is also possible that SU(HW) is only important at LAD borders in specific cell types.

### Effects on gene regulation?

Given the strong overall repression of genes in LADs, together with observations that the NL can actively contribute to gene silencing [14], [49], [50], it may be expected that the effects of SU(HW) on genome – NL interactions also have impact on gene repression in LADs. Interestingly, gypsy elements, which harbor strong SU(HW) binding sites, can boost transgene expression at many integration sites [51]. Possibly the antagonistic action of SU(HW) on NL interactions contributes to this anti-silencing effect. However, analysis of microarray expression data after SU(HW) knockdown or overexpression in Kc cells (data not shown) did not reveal preferential misregulation of genes in SU(HW)-marked LADs or genes that have respectively increased or decreased levels of NL interactions. Redundant mechanisms may provide robustness to gene repression in LADs and may thus mask a contribution of SU(HW). This notion is supported by the fact that *su(Hw)* mutant flies are viable and exhibit a phenotype that is restricted to impaired oogenesis (Phenotypic Descriptions of Classical Alleles from http://flybase.org/reports/FBgn0003567.html). Finally, it should be considered that the effects of SU(HW) on genome – NL interactions, and thus on the spatial organization of interphase chromosomes, may be important for other nuclear processes, such as the regulation of DNA replication or repair [52].

## Materials and Methods

### Constructs

Dam-LAM (pDamMyc-Dm_0_), Dam-Gaf (pNDamMyc-Gaf) and control Dam-only (pNDamMyc) constructs have been described previously [6], [7], [34]. To obtain pGWNDamMyc-su(Hw), pGWNDamMyc-CTCF, and pGWC-Dwg-MycDam, the open reading frames were amplified from cDNA clones (LD15893, GH14774, LD44361, LD45751) and cloned in-frame with Dam by using TOPO cloning and GATEWAY recombination as described [30]. Dam-su(Hw) and Dam-Beaf32 were published before [33].

For overexpression of SU(HW) we constructed vector pA-su(Hw)-iresR, and as a control pAWiresR. To obtain pAWiresRFP, the internal ribosome entry site (IRES) from pUAST-3xEGFP [53] was amplified using primers that incorporate a STOP codon just 5′ of the IRES, and SacI sites at both ends of the amplicon. This fragment was cloned into the SacI site of pAWR (The *Drosophila* Gateway^a^ Vector Collection, a resource developed by the Murphy lab, Carnegie Institute) to yield pAWiresR. pA-su(Hw)-iresR was obtained by GATEWAY recombination with pAWiresR.

### DamID

DamID was performed as described before [54]. In brief, Dam-fusion and Dam-only expression vectors were transfected in parallel into separate dishes of Kc cells by electroporation. Genomic DNA was isolated after 24 h and adenine-methylated fragments were amplified from genomic DNA by methylation-specific PCR. 1 µg of amplified methylated DNA was labeled with Cy-dye labeled random nonamers (TriLink Biotechnologies, according to NimbleChip Arrays User's Guide: ChIP-chip Analysis v2.0). To correct for nonspecific binding of Dam and local differences in DNA accessibility, methylated fragments of Kc cells transfected with a Dam-only construct were labeled with a different fluorescent dye. 13 µg of labeled Dam-fusion and 13 µg of Dam-only methylated fragments were pooled and hybridized to microarrays carrying 380,000 60-mer DNA oligonucleotides [55] (Roche-NimbleGen). Median probe spacing is 300 bp. For each profile, material from two independent experiments was hybridized in opposite dye orientations over Dam controls. The obtained Dam-fusion/Dam-only ratio reflects the extent of protein binding to each fragment on the microarray, corrected for local differences in chromatin accessibility. Probes are mapped to *Drosophila melanogaster* genome sequence release 4.3.

### Knockdown and overexpression of SU(HW)

NL interaction profiles after knockdown of SU(HW) were obtained by using dsRNAs directed against *white* and *su(Hw)*. dsRNAs were *in vitro* transcribed using the RiboMax kit (Promega) from PCR amplicons. PCR amplicons were designed according to the Harvard *Drosophila* RNAi Screening Centre (www.flyrnai.org; *su(Hw)* HFA17074 and MRC020_B05), or as published before for *white* [56]. RNAi treatments were performed as described before [30], with the exception that the treatment was repeated at day 5 and cells were transfected with DamID constructs on day 7.

NL interaction profiles after overexpression of SU(HW) were obtained by co-transfection of DamID vectors and respective overexpression vectors. For overexpression of SU(HW) we used vector pAsu(Hw)iresR. As a control we used the vector pAWiresR. Genomic DNA was isolated after 48 h instead of 24 h. Expression levels of SU(HW) were monitored with Western blot analysis, presenting the protein expression level within the entire cell population. However, because typically 20–30% of cells are transfected, this yields an underestimate of the degree of overexpression.

### SU(HW) antibody

The antibody against the C-terminal peptide of SU(HW) was kindly provided by P. Geyer [57].

### Expression analysis

Total RNA was extracted with TRIzol (Invitrogen) and treated with DNaseI. Isolated RNA from three independent cell cultures was labeled with Cy5 and with Cy3 and co-hybridized to INDAC oligo arrays (http://www.indac.net) printed at the NKI Central Microarray Facility, with each oligonucleotide spotted twice. Raw data from three biological replicates were loess normalized per subarray, and averaged. A-values, (log_2_(Cy5)+log_2_(Cy3))/2, were used for further analysis.

### Data analysis

Microarray data analysis was performed with R [58]. Raw data from two biological replicates were loess normalized, median centered, and dye swap arrays were averaged. For the NL interaction profile after SU(HW) knockdown, the normalized data from the different dsRNA amplicons were averaged as well. To calculate the correlation with previously published low resolution data, the high resolution data were re-sampled to the resolution of the published cDNA microarrays by averaging values for probes from the high resolution array whose center falls within the space of one probe of the cDNA array.

LADs were defined as described in [8]. In short; sharp transitions in the DamID signal were identified using a sliding edge filter (window size 199 probes), and adjacent transitions exceeding a threshold (here 0.3) were combined into domains if at least 70% of the enclosed probes have a positive log_2_ ratio. Polycomb domains were taken from [37] and transposed to FlyBase release 4. Insulator peak positions were determined as follows: after applying a running mean of 5 probes, the derivative was calculated over the running-mean with a 7 probe window. In addition FDR-corrected p-values were determined for each probe using linear modeling (LiMMA) [59]. Peaks were assigned at transitions of the derivative from a positive to a negative value (indicating a peak) and where in addition at least three probes were significantly enriched (p<0.005). Motif scans were performed using the TFBS Perl module [60] with position weight matrices (PWMs) obtained from literature [38], [61] and the TRANSFAC database [62]. Briefly, PWMs were compared against the genomic sequence and a relative matching score was calculated based on a PWM's information content. A matching score of 85% (CTCF, SU(HW)) and 99% (GAF) was used as it yielded a similar number of matches to the identified in vivo binding sites. Custom R scripts were used to align data to LAD borders or to SU(HW) peaks; for this purpose, genome-wide positions of all analyzed features were converted to coordinates relative to the nearest border or peak. In case of LADs, data around right-side borders were mirrored and combined with data around left-side borders. The region around borders from which data was taken ranges from the middle of inter-LAD regions to the middle of LADs themselves; this ensures that all datapoints are used only once. Similarly, in case of alignments to Su(HW) peaks, the region ranged halfway to the next peak. Median binding ratios across the aligned borders or peaks were calculated with a running window covering 5% of the data within the aligned region for alignments at LADs (Fig. 1def, Fig. 4bc, Fig. 5ab), 20% and 10% for changes in NL interaction (Fig. 5c) and 10% for aligning at Su(Hw) peaks (Fig. 5d,e).

### Data availability

DamID and expression data have been deposited in NCBI's Gene Expression Omnibus and are accessible through GEO Series accession number GSE20313.

## Supporting Information

Figure S1 **Genome - NL interaction map in *Drosophila* Kc cells on all chromosomes**. Y-axes depict the log_2_ transformed Dam-LAM over Dam-only methylation ratio with a running median of 15 probes. Red rectangles represent LADs.
(TIF)

Figure S2 **Insulator protein binding map in *Drosophila* Kc cells** (**A**) Insulator protein binding maps at four arbitrarily chromosomal regions. Y-axes depict the Dam-insulator over Dam-only methylation ratio. Grey rectangles represent LADs. (**B**) Co-occurrence of insulator proteins indicated by a density plot of the log2 transformed binding ratio of each insulator protein (colored lines) at the binding peaks of each insulator protein (different panels).
(TIF)

Figure S3 **DamID maps are consistent with ChIP and sequence motif distributions.** (**A**) Binding maps of BEAF-32, GAF, CTCF and SU(HW) at random regions of chromosome 2L for Dam-insulator over Dam-only methylation ratios (colored lines) versus ChIP scores (black). (**B**) DamID binding maps of CTCF and SU(HW) (colored lines) at chromosome 2L versus the location of corresponding sequence motifs (black).
(TIF)

Figure S4 **No SU(HW) enrichment in Polycomb domains.** (**A**) Binding maps of insulator proteins along a five sequential 1Mb regions at chromosome 2L. Y-axes depict the linear Dam-SU(HW) over Dam-only methylation ratio. Grey rectangles represent the Polycomb domains.
(TIF)

Figure S5 **No preferential spacing of SU(HW) peaks in a range of 40kb.** Histogram of the pair-wise distances between all SU(HW) peaks. X-axis depicts genomic distance between the peaks.
(TIF)

Figure S6 **Changes in NL interaction after altering SU(HW) expression levels are reproducible.** Ave changes in NL interaction levels per LAD, for LADs without SU(HW) (grey), LADs with at least one SU(HW) peak (light blue or orange), the 25% of LADs with the highest SU(HW) peak density (dark blue or orange) after knockdown with amplicon 1 (blue, upper panles), knockdown with amplicon 2 (blue, middle panels) and overexpression of SU(HW) (orange, lower panels). First experiment (left panels), second experiment (right panels).
(TIF)

Table S1 **LAD positions.** This file is a flat text file in GFF format (http://www.sanger.ac.uk/Software/formats/GFF) listing the positions of all 412 LADs (*Drosophila melanogaster* genome sequence release 4.3). Score (column 6) indicates the fraction of array probes inside the LAD with a positive LAM DamID logratio, after applying a running median filter with window size 5.
(TXT)
