# Supplementary figures and images for: The Insulator Protein SU(HW) Fine-Tunes Nuclear Lamina Interactions of the Drosophila Genome
Source: PLoS One. 2010 Nov 24;5(11):e15013. doi: 10.1371/journal.pone.0015013 (PMC2991331; doi:10.1371/journal.pone.0015013)

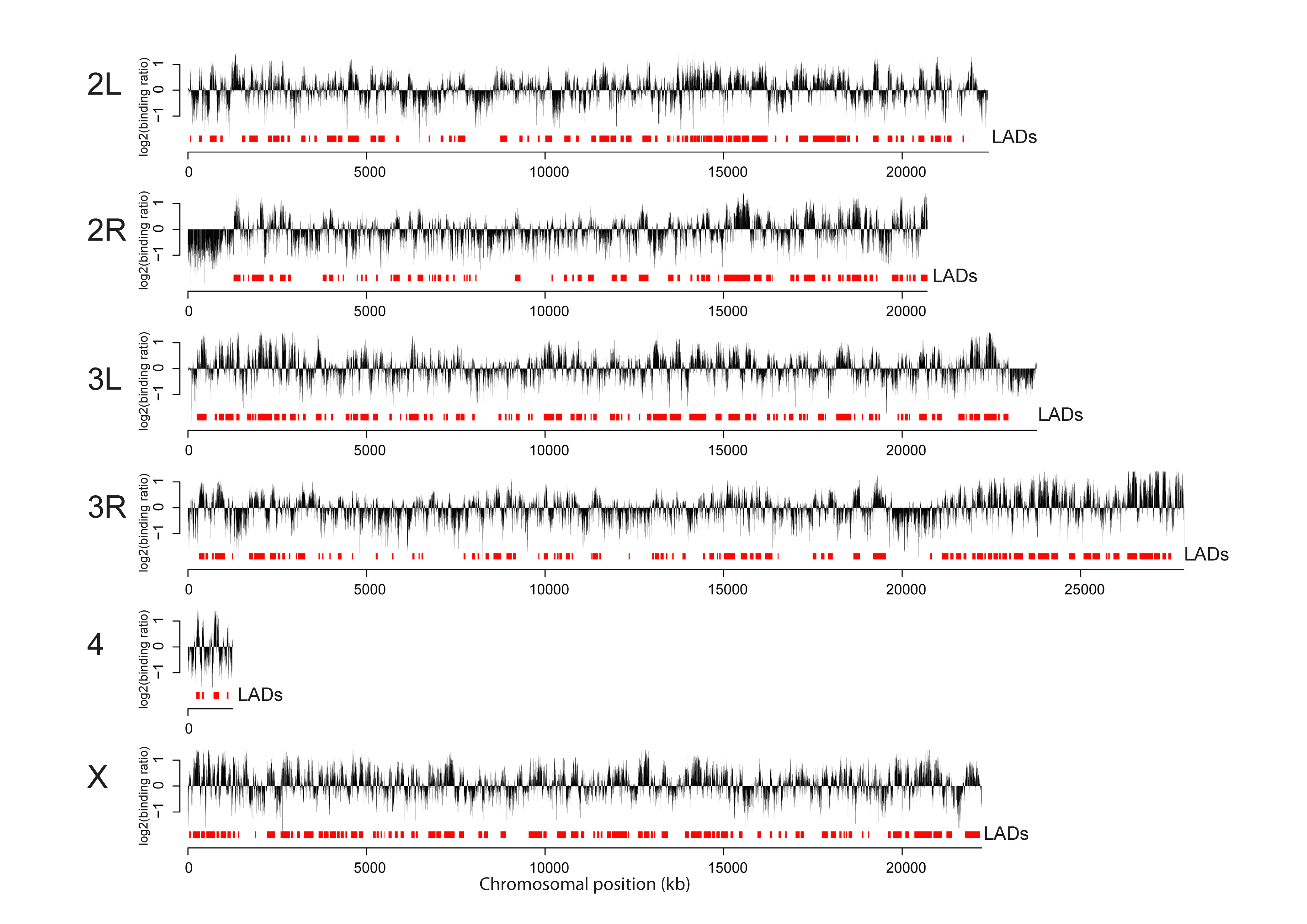

Supplement: Figure S1 — Genome - NL interaction map in Drosophila Kc cells on all chromosomes. Y-axes depict the log2 transformed Dam-LAM over Dam-only methylation ratio with a running median of 15 probes. Red rectangles represent LADs. (TIF) [file pone.0015013.s001.tif]

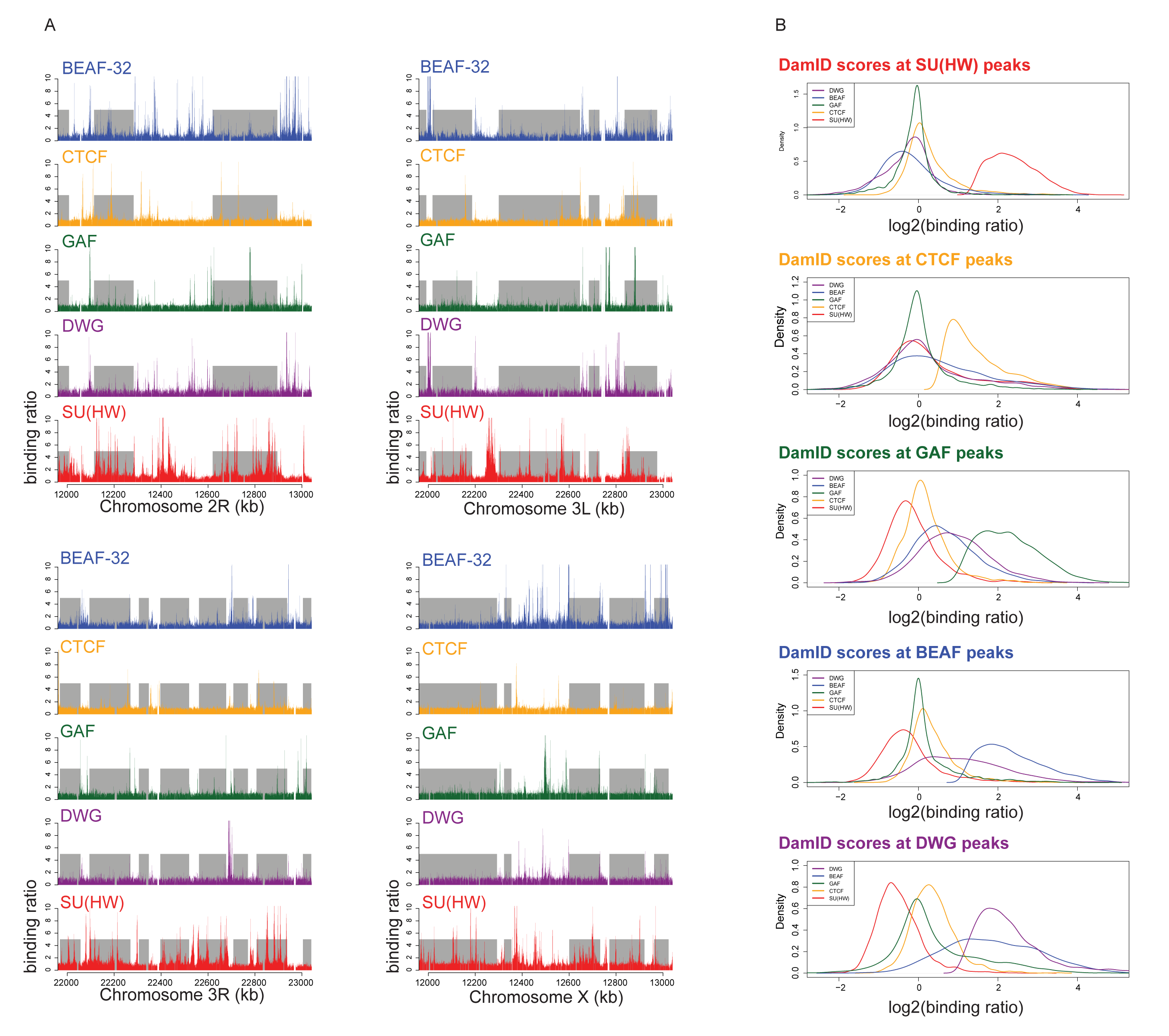

Supplement: Figure S2 — Insulator protein binding map in Drosophila Kc cells (A) Insulator protein binding maps at four arbitrarily chromosomal regions. Y-axes depict the Dam-insulator over Dam-only methylation ratio. Grey rectangles represent LADs. (B) Co-occurrence of insulator proteins indicated by a density plot of the log2 transformed binding ratio of each insulator protein (colored lines) at the binding peaks of each insulator protein (different panels). (TIF) [file pone.0015013.s002.tif]

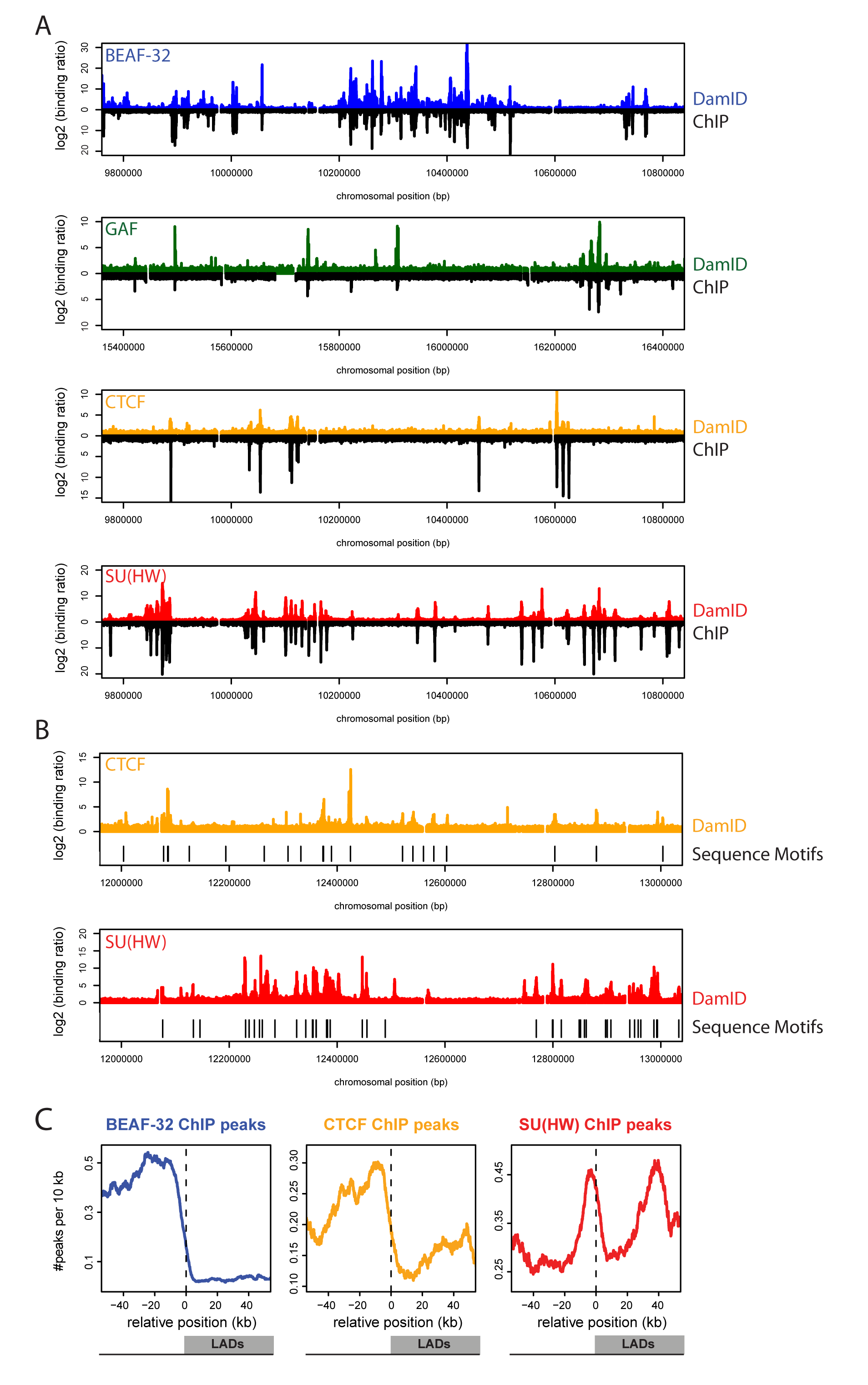

Supplement: Figure S3 — DamID maps are consistent with ChIP and sequence motif distributions. (A) Binding maps of BEAF-32, GAF, CTCF and SU(HW) at random regions of chromosome 2L for Dam-insulator over Dam-only methylation ratios (colored lines) versus ChIP scores (black). (B) DamID binding maps of CTCF and SU(HW) (colored lines) at chromosome 2L versus the location of corresponding sequence motifs (black). (TIF) [file pone.0015013.s003.tif]

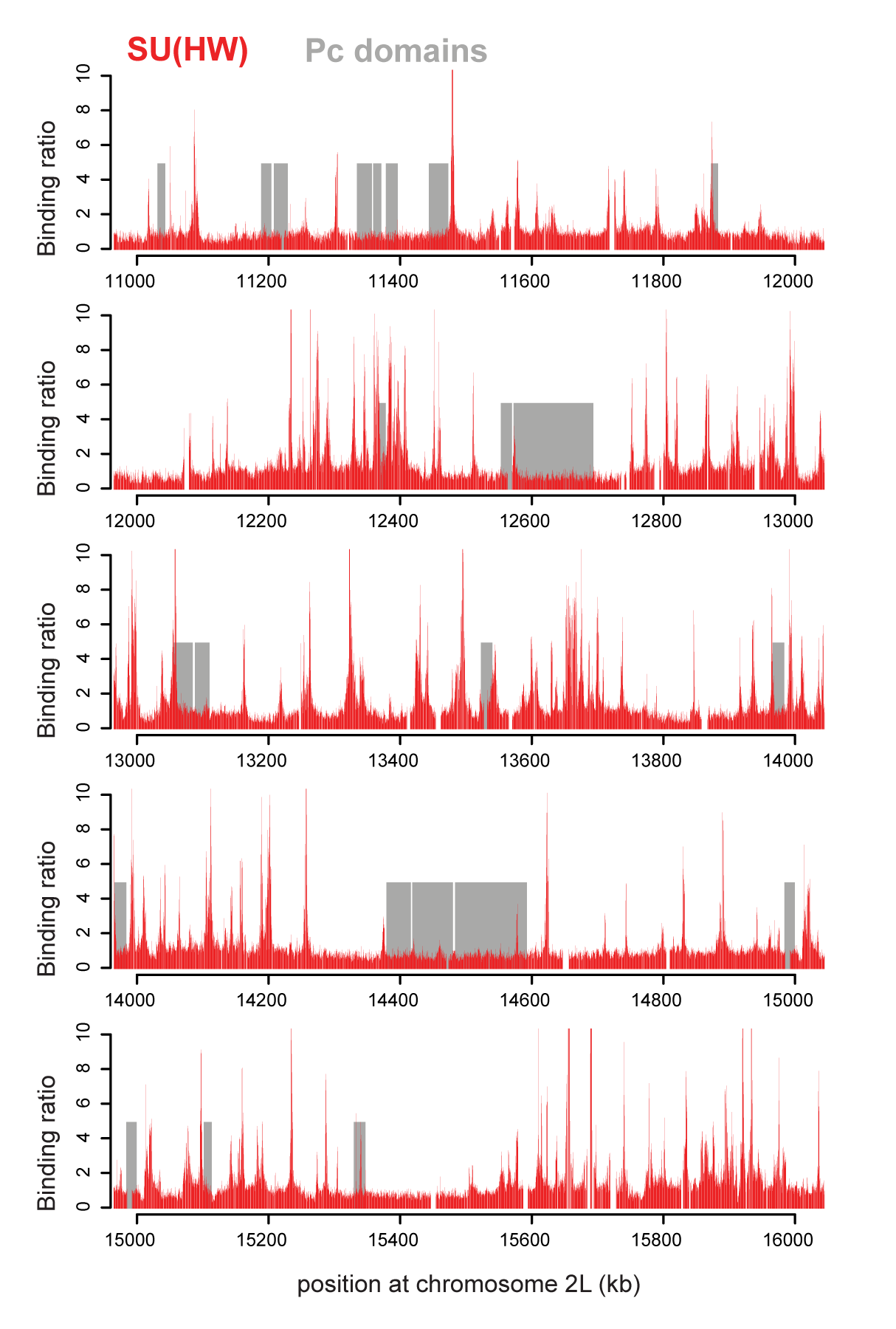

Supplement: Figure S4 — No SU(HW) enrichment in Polycomb domains. (A) Binding maps of insulator proteins along a five sequential 1Mb regions at chromosome 2L. Y-axes depict the linear Dam-SU(HW) over Dam-only methylation ratio. Grey rectangles represent the Polycomb domains. (TIF) [file pone.0015013.s004.tif]

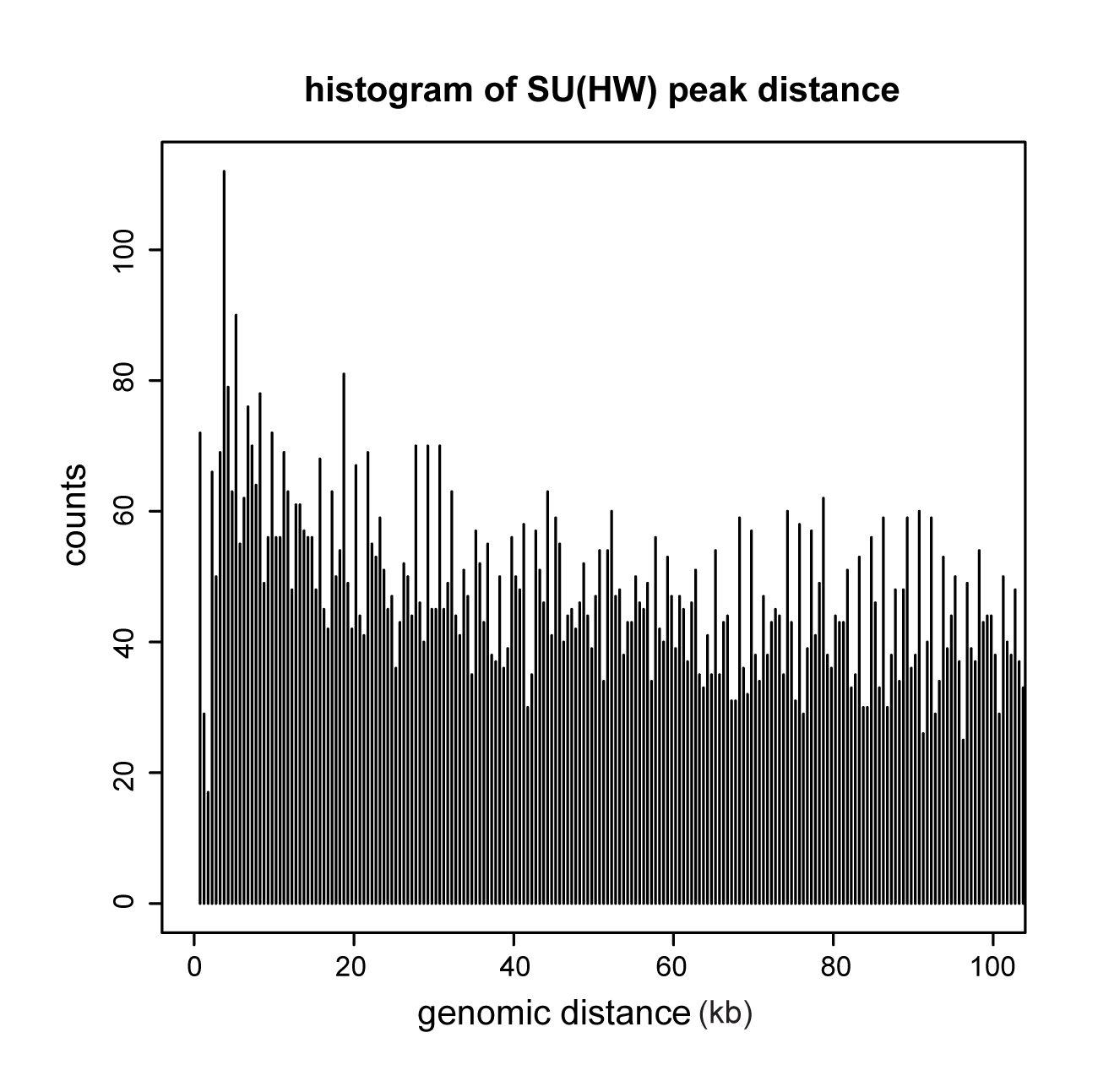

Supplement: Figure S5 — No preferential spacing of SU(HW) peaks in a range of 40kb. Histogram of the pair-wise distances between all SU(HW) peaks. X-axis depicts genomic distance between the peaks. (TIF) [file pone.0015013.s005.tif]

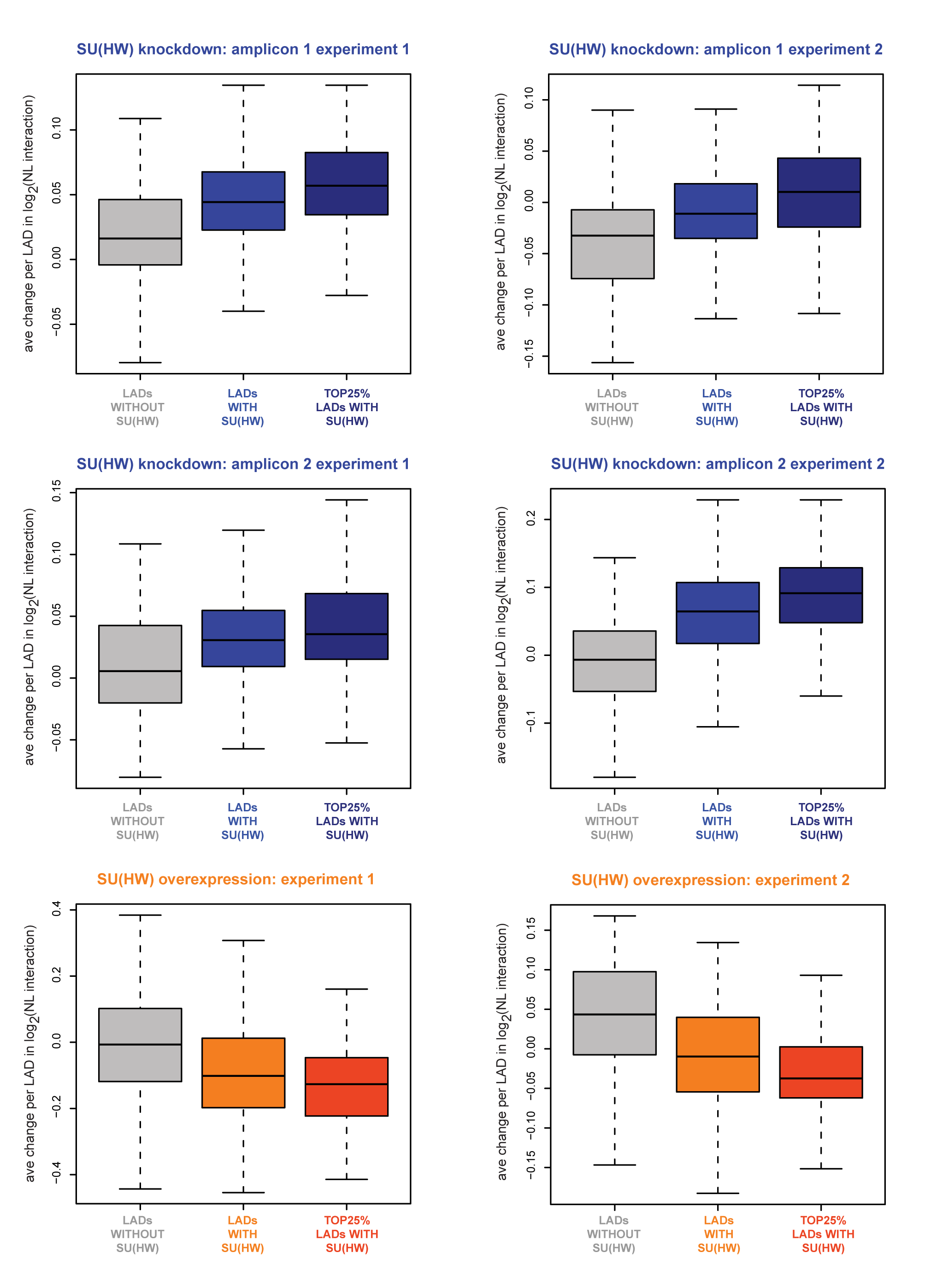

Supplement: Figure S6 — Changes in NL interaction after altering SU(HW) expression levels are reproducible. Ave changes in NL interaction levels per LAD, for LADs without SU(HW) (grey), LADs with at least one SU(HW) peak (light blue or orange), the 25% of LADs with the highest SU(HW) peak density (dark blue or orange) after knockdown with amplicon 1 (blue, upper panles), knockdown with amplicon 2 (blue, middle panels) and overexpression of SU(HW) (orange, lower panels). First experiment (left panels), second experiment (right panels). (TIF) [file pone.0015013.s006.tif]
